# Performance of Surgical Stress Index during Sevoflurane-Fentanyl and Isoflurane-Fentanyl Anesthesia

**DOI:** 10.1155/2010/810721

**Published:** 2010-04-06

**Authors:** S. Mustola, T. Parkkari, K. Uutela, M. Huiku, M. Kymäläinen, J. Toivonen

**Affiliations:** ^1^Department of Anesthesia, South Carelia Central Hospital, Valtokäkelänkatu 1, 53130 Lappeenranta, Finland; ^2^Clinical Research, GE Healthcare Finland Oy, P.O. Box 900, 00031 GE Helsinki, Finland

## Abstract

The performance of recently introduced Surgical Stress Index (SSI), based on heart rate and photoplethysmography, was estimated during sevoflurane-fentanyl and isoflurane-fentanyl anesthesia during surgical procedures. Forty ASA I–III patients were enrolled. Anesthesia was induced with fentanyl 2 *μ*g kg^−1^ and thiopentone 3–5 mg kg^−1^. Tracheal intubation was performed 5 minutes after fentanyl bolus. Patients were randomly allocated to receive sevoflurane (*n* = 20) or isoflurane (*n* = 20) in 30% oxygen/air. State entropy was kept at 40–60, target being 50. During surgery, fentanyl boluses 1.5 *μ*g kg^−1^ were given at 30–40-minute intervals. SSI increased significantly after intubation. During surgery, the decrease of SSI after fentanyl boluses was similar in sevoflurane and isoflurane groups but SSI values were higher in sevoflurane than in isoflurane group. Tracheal intubation, skin incision, and surgical stimuli increased SSI from baseline, indicating that nociceptive stimuli increase SSI. Fentanyl boluses during surgery decreased SSI, indicating that increasing analgesia decreases SSI.

## 1. Introduction

Estimating nociception during general anesthesia is very challenging since there have not been any direct methods to measure it. That is why clinical evaluation of nociception is based on unspecific autonomic reactions, such as blood pressure, heart rate, sweating, or tearing. Also determination of plasma concentrations of opioids (directly or estimated by pharmacokinetic models) has been used [1]. Clinical end-points, such as movement reaction in response to nociceptive stimulus, can be used as an indicator of inadequate analgesia [2], but it is not very useful during operation when patients are paralysed. Some electroencephalographic (EEG)-derived parameters, such as Entropy, are suggested to reflect also the nociceptive component of anesthesia [3–5]. Changes in skin conductivity and suppression of photoplethysmographic pulse wave amplitude (PPWA) have also been proposed as indicators of nociception [6, 7]. Recently, Surgical Stress Index (SSI), based on a sum of normalized pulse beat interval (PBI) and PPWA, was introduced for the assessment of surgical stress or nociception [8]. PBI and PPWA were combined and a simple numerical index (from 0 to 100) suitable for monitoring surgical stress was developed. Huiku et al. showed that SSI is high when noxious stimulation is high or the remifentanil concentration inadequate and that SSI is low when remifentanil concentration is high or the stimulation low [8]. SSI has shown to be a better measure of nociception than entropy parameters, heart rate, or PPWA [9]. 

Evaluation of SSI has been accomplished during experimental or standardized situations. Our purpose was to assess the function of SSI in clinical situation. We recorded SSI values during sevoflurane-fentanyl and isoflurane-fentanyl anesthesia with special attention to tracheal intubation, skin incision, and effect of fentanyl boluses during surgery. 

## 2. Materials and Methods

The study was approved by the local institutional ethics committee (South Carelia Central Hospital, Lappeenranta, Finland), and written informed consent was obtained from all patients. We enrolled forty patients (Table 1), ASA I–III, scheduled for surgical procedure under general anesthesia. Exclusion criteria were known heart arrhythmia (such as chronic atrial fibrillation), neurological disorder, medication affecting central nervous system, and history of alcohol or drug abuse. The ECG, photoplethysmography, peripheral oxygen saturation (SpO_2_), noninvasive blood pressure (NIBP), end-tidal CO_2_, minimal alveolar concentrations (MAC) of sevoflurane or isoflurane, and entropy parameters [state entropy (SE) and response entropy (RE)] were monitored and collected using a data acquisition PC (Datex-Ohmeda S/5 Anesthesia Monitor, S/5 iCentral Network Workstation and S/5 iCollect data acquisition software, GE Healthcare Finland Oy, Helsinki, Finland). To collect user feedback, the SSI was visible on the data acquisition PC during the procedure. However, the SSI values used for statistical analysis were calculated offline with the algorithm described by Huiku et al. [8]. SSI is calculated as SSI = 100 − (0.33 × PBI + 0.67 × PPWA). The SSI values at baseline before induction of anesthesia are mostly 60–70, after induction of anesthesia 20–40 (without stimuli), after tracheal intubation 50–70, and during surgery 50–70 [8, 9]. 

Patients, premedicated with oral diazepam 0.1 mg kg^−1^ adjusted to the nearest 2.5 mg, received fentanyl 2 *μ*g kg^−1^ and thiopentone 3–5 mg kg^−1^ for induction of anesthesia. Rocuronium 0.6 mg kg^−1^ was given to facilitate tracheal intubation. Tracheal intubation was performed 5 minutes after fentanyl bolus. Patients were randomly assigned to two groups sevoflurane (SEVO, *n* = 20) and isoflurane (ISOF, *n* = 20) in 30% oxygen in air as a maintenance inhalation agent during anesthesia. After tracheal intubation sevoflurane or isoflurane were adjusted to maintain state entropy (SE) level between 40 and 60 target being 50. During procedure, fentanyl boluses 1.5 *μ*g kg^−1^ were given repeatedly at rather long 30–40-minute intervals to enhance the variability in its effects during the procedure. Reactivity of SSI to tracheal intubation, surgery, and fentanyl boluses was recorded. The average SSI response to fentanyl bolus during procedure was quantified by comparing the average SSI level 1–5 minutes before the bolus to the average SSI level 4–8 minutes after the bolus [time to peak effect for fentanyl has been reported to be 4–6 minutes [10]]. If mean arterial pressure (NIBP_MAP_) decreased below 60 mmHg, ethylphenylephrin 2 mg was given intravenously. Our test hypothesis was that the SSI would increase after the intubation and skin incision and that the fentanyl boluses would decrease the SSI.

### 2.1. Statistics

The statistical analysis was performed with the SPSS program (SPSS 14.0 for Windows, SPSS Inc., Chicago, IL, USA). Wilcoxon Signed Ranks test was used to compare changes inside groups and two-tailed Mann-Whitney *U* test for between groups changes; *P* < .05 was considered statistically significant. Values are presented as mean ± SD unless otherwise specified.

## 3. Results

**Figure 1:**
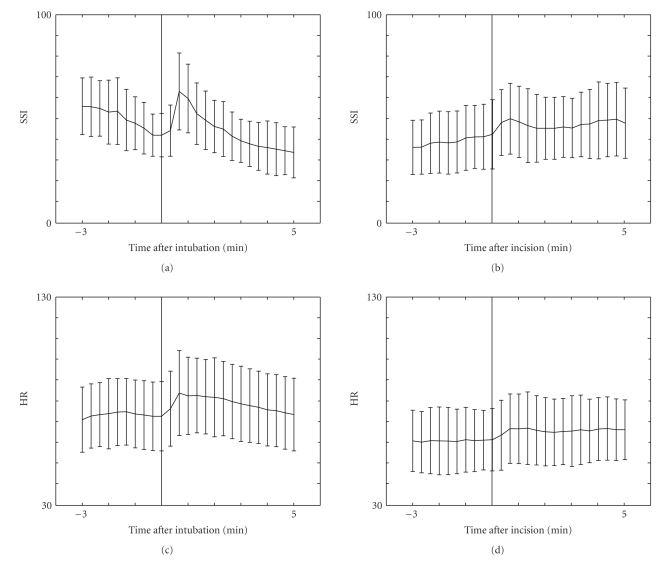
Mean surgical stress index (SSI ± SD) and heart rate (HR ± SD) values before and after tracheal intubation and skin incision.

**Figure 2:**
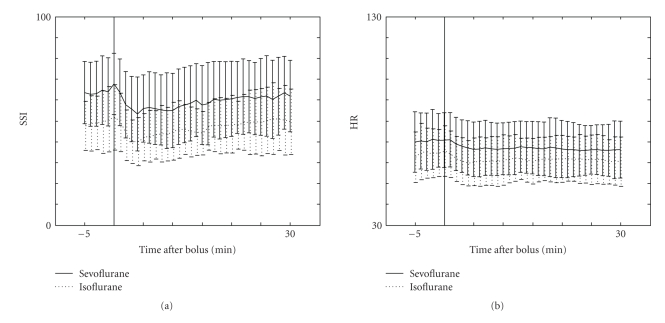
Mean surgical stress index (SSI ± SD) and heart rate (HR ± SD) values before and after fentanyl bolus (1.5 *μ*g kg^−1^) during surgery in sevoflurane (*n* = 20) and isoflurane (*n* = 20) groups.

The patient characteristics and surgical procedures in the two groups were similar (Table 1). The values of SSI, HR, NIBP_MAP_, SE, and RE at different time points are presented in Table 2. After tracheal intubation, both SSI and HR increased significantly (*P* < .001, Figure 1) but after skin incision only SSI increased significantly (*P* < .001, Figure 1). The decrease of SSI during procedure after fentanyl bolus was 7.5 ± 15.0, (*P* < .01). The decrease of SSI was similar in both SEVO and ISOF groups but SSI values in SEVO group were higher than in ISOF group during surgery (Figure 2). MAC and SE values were similar in SEVO and ISOF groups before fentanyl boluses [0.74 ± 0.12 versus 0.77 ± 0.17, (*P* = .32) and 43.5 ± 8.1 versus 45.2 ± 8.3, (*P* = .47), resp.]. However, HR values were significantly different in SEVO and ISOF groups 70.6 ± 13.0 versus 64.9 ± 11.8, (*P* < .01), respectively (Figure 2). After fentanyl boluses, SE and HR changes were similar and MAC values remained stable in both groups.

## 4. Discussion

We evaluated the performance of SSI during sevoflurane-fentanyl and isoflurane-fentanyl anesthesia in diverse surgical procedures. According to our hypothesis, the intubation and skin incision increased SSI and the fentanyl boluses decreased it. Fentanyl bolus 2 *μ*g kg^−1^ given five minutes before tracheal intubation did not block the haemodynamic or SSI response to intubation. 

During procedure fentanyl boluses, 1.5 *μ*g kg^−1^ given at 30–40-minute intervals caused equal decrease of SSI in sevoflurane and isoflurane groups but absolute SSI values were different between groups. We examined MAC, SE, RE, and haemodynamic variables and we noticed that HR values were different between groups but the other variables were similar. Heart beat interval accounts 33% of the absolute value of SSI and 67% of it consist of PPWA [8]. That is why it is logical that if anesthetics have different effects on HR then there are also different absolute values of SSI. It has been noticed in an earlier study that desflurane increases heart rate and prolongs the ECG QT interval more than sevoflurane [11]. On the other hand, Yildirim et al. [12] found that desflurane, sevoflurane, and isoflurane all prolonged QT interval but there were no between group differences. They also found that HR was higher in sevoflurane group 3 minutes after intubation compared to isoflurane and desflurane groups, but 10 minutes after reaching 1 MAC steady state concentration, there were no between group differences [12]. It has been shown that during sevoflurane or isoflurane anesthesia, there can be bradycardia episodes [13]. However, different HR values explain at least partly the different SSI values during procedures. This means that when one interprets absolute SSI values, the effect of different anesthetics on HR must be taken into consideration. 

Seitsonen et al. [7] showed that HR changes offer some information on adequacy of analgesia at skin incision during sevoflurane anesthesia, but suggested that multiparameter approach is required for accurate monitoring of nociception. Huiku et al. [8] developed the multivariate SSI, and in a validation study they found that SSI was accurate measurement of the nociception—antinociception balance in response to skin incision and surgery during propofol—remifentanil anesthesia. Struys et al. [9] showed that HR had some value in estimating analgesia during propofol-remifentanil anesthesia, but it was influenced by the hypnotic component of anesthesia while SSI was not. They concluded that SSI seems to be better than SE, RE, HR, or PPGA when correlating nociceptive reactions to remifentanil concentrations and that SSI might be more accurate for nociception. Our results confirm that SSI acts consistently at nociceptive situations (tracheal intubation, skin incision, surgery). 

Fentanyl bolus (2 *μ*g kg^−1^), given five minutes before tracheal intubation, did not block the SSI response. Although we did not have any placebo group, we can assume that the reaction would have been more pronounced without fentanyl. After all, there were some patients whose reaction measured by SSI was minimal but in some other patients it was very clear. We think that this is due to interindividual difference in responsiveness and differences in opioid (or other drugs) requirements. 

Our results were calculated off line [8]. The SSI was also continuously calculated and displayed on line during the study in a data acquisition PC using basically the same algorithm with slight modifications needed for the PC version. This version of SSI showed some increases with no obvious exceptionally nociceptive stimulation. However, it is rather difficult to evaluate the exact level of nociception. For example, during laparothomy it cannot always be seen when the gut is distended or not by surgeon. In further studies, one solution for this could be video camera; then you can see all the time what the surgeon is doing. 

Especially in older patients we sometimes, before the procedure was started or if there was a break in surgery, observed inconsistency between blood pressure and SSI: while NIBP_MAP_ decreased below 60 mmHg, SSI reported poor signal logically and increased. When ethylphenylephrin 2 mg was given iv., NIBP_MAP_ increased and SSI decreased. In some younger patients, SSI was stable and the signal was good even when NIBP_MAP_ was below 60 mmHg. Therefore, at least in older patients when NIBP_MAP_ decreased below 60 mmHg, SSI acted like increased nociception and after ethylphenylephrin it acted like decreased nociception. 

In two neck-surgery patients, SSI increased remarkably when surgeon palpated the thyroid gland area before skin incision. This can be due to sensitive baroreflex reaction or maybe the palpation moved also tracheal tube and then the stimulus can be rather strong. 

## 5. Conclusions

Fentanyl bolus (2.0 *μ*g kg^−1^), given five minutes before tracheal intubation, did not block totally the increase of SSI. Fentanyl boluses during procedure decreased SSI, indicating that increasing analgesia decreases SSI. Tracheal intubation, skin incision, and surgical stimuli during the procedure increased SSI from baseline, indicating that nociceptive stimuli increase SSI. We noticed some problems in this version of SSI but it seems that SSI is a valuable tool in estimating nociception-antinociception balance during anesthesia. However, more studies are needed to evaluate its final value.

## Figures and Tables

**Table 1:** Patient characteristics. Values are mean (±SD).

	Sevoflurane	Isoflurane
	(*n* = 20)	(*n* = 20)
Gender (Male/Female)	10/10	5/15
ASA I/II/III	4/12/4	4/13/3
Age (years)	53 (17)	58 (13)
Height (cm)	170 (7)	166 (7)
Weight (kg)	70 (14)	74 (14)

ASA: American society of anesthesiologists physical status.

**Table 2:** Mean surgical stress index (SSI ± SD), heart rate (HR ± SD), noninvasive mean arterial pressure (NIBP_MAP_± SD), spectral entropy (SE ± SD), and response entropy (RE ± SD) values at different measuring points.

	SSI	HR	NIBP_MAP_	SE	RE
Baseline	60.3 (9.6)	71.1 (11.9)	108.9 (14.1)	86.4 (6.2)	95.5 (6.0)
Before intubation	44.2 (9.9)	73.2 (15.7)	95.0 (13.9)	60.3 (14.4)	62.6 (15.2)
After intubation	61.5 (12.9)^#^	82.3 (17.8)^#^	106.7 (23.4)^#^	68.8 (12.8)^#^	72.6 (13.6)^#^
Before incision	38.1 (12.4)	61.3 (14.5)	68.9 (9.9)	46.7 (8.9)	48.9 (9.3)
After incision	47.5 (13.3)^#^	66.4 (15.6)	81.9 (17.5)^#^	45.3 (10.0)	47.3 (10.7)

#: statistically significant increase of different values compared to the value in the former square.

## References

[1] Bouillon TW, Bruhn J, Radulescu L (2004). Pharmacodynamic interaction between propofol and remifentanil regarding hypnosis, tolerance of laryngoscopy, bispectral index, and electroencephalographic approximate entropy. *Anesthesiology*.

[2] Zbinden AM, Maggiorini M, Petersen-Felix S, Lauber R, Thomson DA, Minder CE (1994). Anesthetic depth defined using multiple noxious stimuli during isoflurane/oxygen anesthesia: I. Motor reactions. *Anesthesiology*.

[3] Vakkuri A, Yli-Hankala A, Talja P (2004). Time-frequency balanced spectral entropy as a measure of anesthetic drug effect in central nervous system during sevoflurane, propofol, and thiopental anesthesia. *Acta Anaesthesiologica Scandinavica*.

[4] Viertiö-Oja H, Maja V, Särkelä M (2004). Description of the Entropy^TM^
algorithm as applied in the Datex-Ohmeda 5/5^TM^
Entropy Module. *Acta Anaesthesiologica Scandinavica*.

[5] Takamatsu I, Ozaki M, Kazama T (2006). Entropy indices vs the bispectral index for estimating nociception during sevoflurane anaesthesia. *British Journal of Anaesthesia*.

[6] Luginbüh M, Reichlin F, Sigurdsson GH, Zbinden AM, Petersen-Felix S (2002). Prediction of the haemodynamic response to tracheal intubation: comparison of laser-Doppler skin vasomotor reflex and pulsed wave reflex. *British Journal of Anaesthesia*.

[7] Seitsonen ERJ, Korhonen IKJ, van Gils MJ (2005). EEG spectral entropy, heart rate, photoplethysmography and motor responses to skin incision during sevoflurane anaesthesia. *Acta Anaesthesiologica Scandinavica*.

[8] Huiku M, Uutela K, van Gils M (2007). Assessment of surgical stress during general anaesthesia. *British Journal of Anaesthesia*.

[9] Struys MMRF, Vanpeteghem C, Huiku M, Uutela K, Blyaert NBK, Mortier EP (2007). Changes in a surgical stress index in response to standardized pain stimuli during propofol-remifentanil infusion. *British Journal of Anaesthesia*.

[10] Scott JC, Cooke JE, Stanski DR (1991). Electroencephalographic quantitation of opioid effect: comparative pharmacodynamics of fentanyl and sufentanil. *Anesthesiology*.

[11] Silay E, Kati I, Tekin M (2005). Comparison of the effects of desflurane and sevoflurane on the QTc interval and QT dispersion. *Acta Cardiologica*.

[12] Yildirim H, Adanir T, Atay A, Katirciolu K, Savaci S (2004). The effects of sevoflurane, isoflurane and desflurane on QT interval of the ECG. *European Journal of Anaesthesiology*.

[13] Godet G, Watremez C, El Kettani C, Soriano C, Coriat P (2001). A comparison of sevoflurane, target-controlled infusion propofol, and propofol/isoflurane anesthesia in patients undergoing carotid surgery: a quality of anesthesia and recovery profile. *Anesthesia and Analgesia*.

